# Double‐bundle versus single‐bundle medial patellofemoral ligament reconstruction for recurrent patellar dislocation: A meta‐analysis

**DOI:** 10.1002/jeo2.70112

**Published:** 2024-12-18

**Authors:** Yiheng Wu, Junran Li, Hongbo Zhao, Hongyan Zhou, Bokai Wang, Jinlong Zhang, Shengkun Zhao

**Affiliations:** ^1^ Department of Orthopedics, The Second Hospital of Tangshan Graduate School of North China University of Science and Technology Tangshan China; ^2^ Department of Orthopedics The Second Hospital of Tangshan Tangshan China; ^3^ Department of Orthopedics Tangshan Central Hospital Tangshan China

**Keywords:** double‐bundle, medial patellofemoral reconstruction, meta‐analysis, recurrent patellar dislocation, single‐bundle

## Abstract

**Purpose:**

To compare the clinical efficacy of single‐bundle versus double‐bundle reconstruction of the medial patellofemoral ligament (MPFL) for recurrent patellar dislocation (RPD) regarding knee function scores, postoperative complications, and imaging assessments.

**Methods:**

A computerized search of PubMed, Cochrane Library, Embase, China Biomedical Literature Database (CBM), China National Knowledge Network (CNKI), and VIP Database was performed for single‐bundle versus double‐bundle reconstruction of the medial patellofemoral ligament for treatment of RPD. Randomized controlled trials (RCTs) were evaluated for quality using the risk‐of‐bias evaluation tool recommended by the Cochrane Collaboration Network, and Cohort studies (CSs) were assessed using the Newcastle‐Ottawa Scale (NOS) scale. Meta‐analysis was performed using RevMan 5.3 software and STATA 16.0.

**Results:**

Thirteen studies were included, four randomized controlled studies, and nine cohort studies. The level of evidence for the four randomized controlled studies was Ⅰ, and the nine cohort studies were Ⅲ. A total of 862 (891 knees) patients were included, of which 448 (465 knees) underwent double‐bundle MPFL reconstruction and 414 (426 knees) underwent single‐bundle MPFL reconstruction. Kujala score (MD = 2.06, 95% confidence interval [CI] [0.11, 4.01], *p* < 0.05), Tegner score (MD = 0.39, 95% CI [0.11, 0.68], *p* < 0.05), International Knee Documentation Committee (IKDC) score (MD = 4.88, 95% CI [1.46, 8.31], *p* < 0.05), and postoperative recurrence instability (odds ratio [OR] = 0.12, 95% CI [0.04, 0.44], *p* < 0.05) were better in the double‐bundle group than in the single‐bundle group. Lysholm score (MD = 0.86, 95% CI [−0.76, 2.48], *p* = n.s), patellar tilt angle (MD = −0.22, 95% CI [−0.54, 0.10], *p* = n.s), patellar lateral shift rate (MD = −0.16, 95% CI [−0.41, 0.09], *p* = n.s), congruence angle (MD = 0.06, 95% CI [−0.41, 0.52], *p* = n.s), postoperative knee pain (OR = 0.39, 95% CI [0.14, 1.11], *p* = n.s), and additional postoperative surgical treatment (OR = 0.20, 95% CI [0.01−6.25], *p* = n.s) had no statistically significant differences.

**Conclusions:**

Double‐bundle reconstruction of the medial patellofemoral ligament for RPD was superior to single‐bundle reconstruction in both knee function scores and postoperative recurrent patellar instability, and double‐bundle reconstruction of the medial patellofemoral ligament for RPD had better clinical outcomes.

**Level of Evidence:**

Level Ⅲ, Ⅰ and Ⅲ studies.

**Abbreviations**
CA: congruence angle
CBM: China Biomedical Literature Database
CI: confidence intervals
CNKI: China National Knowledge Network
CSs: cohort studies
DB: double‐bundle
IKDC: International Knee Documentation Committee
MD: mean differences
MPFL: medial patellofemoral ligament
PLSR: patellar lateral shift rate
PTA: patellar tilt angle
RCTs: randomized controlled trials
RPD: recurrent patellar dislocation
SB: single‐bundle

## INTRODUCTION

Recurrent patellar dislocation (RPD) is related to various pathological abnormalities, mainly due to trochlear dysplasia, tibial tubercle lateralization, and patella alta [4, 7, 28, 29]. However, the most crucial contributing reason for patellar dislocation is injury to the medial patellofemoral ligament (MPFL) [7, 27, 28, 29]. The MPFL, which supplies roughly 50%–60% of the binding force to avoid lateral patellar dislocation during knee flexion, is the most significant soft tissue structure preventing lateral patellar dislocation [13, 21, 26, 32]. It is ruptured in over 90% of cases of first patellar dislocation and almost 100% of cases of re‐dislocation [39]. When it comes to patient satisfaction, quality of life, and knee scores, MPFL reconstruction is seen to be the most effective treatment for RPD. It also has a low incidence of problems and re‐dislocation and produces great results [8, 12, 15, 21].

Single‐bundle (SB) and double‐bundle (DB) reconstruction are the two primary types of MPFL reconstruction. Based on the concept of functional MPFL bundles proposed by Kang et al. [14], featuring an ascending superiorly oblique bundle and a horizontally inferior straight bundle, DB reconstruction of MPFL has attracted increasing interest based on this theory. Other biomechanical studies have demonstrated that DB reconstruction may lead to better clinical results [14, 26, 34]. Nonetheless, some scholars have suggested that the MPFL is a functionally variable fibre complex, with some tension and some laxity in the range of motion of the knee joint [29]. Hence, a reconstruction that mimics the triangular morphology of the MPFL can lead to uneven and nonisometric graft tension, which can produce non‐physiologic patellofemoral loading and kinematics [29]. Originally Gomes et al. [6] reported SB MPFL reconstruction with favorable clinical results. Several more studies have demonstrated that SB MPFL reconstruction yields positive clinical results [7, 17]. Conversely, patellar re‐dislocation occurred in three patients in the SB group and no re‐dislocations in the DB group in a study by Wang et al. [32]. A systematic evaluation by Kang et al. [13] concluded that MPFL reconstruction with the DB technique showed similar results to the SB technique in terms of improvement of knee function, recurrent dislocation, and complications. Regarding SB and DB MPFL reconstruction, there is some controversy, and the majority of studies on MPFL reconstruction have been clinical follow‐up case series with relatively small sample sizes, forcing the use of meta‐analysis.

This meta‐analysis aimed to compare the clinical efficacy of SB versus DB MPFL reconstruction for RPD regarding knee function scores, postoperative complications, and imaging assessments. We hypothesized that DB MPFL reconstruction for the treatment of RPD would have better functional scores, lower postoperative recurrent patellar instability, and the same radiographic findings.

## MATERIALS AND METHODS

The meta‐analysis was carried out according to the Preferred Reporting Items for Systematic Reviews and Meta‐Analyses (PRISMA) statement [23]. This study was registered on PROSPERO (CRD4202349289).

### Search strategy

PubMed, Embase, Cochrane Library, China National Knowledge Network (CNKI), China Biomedical Literature Database (CBM), and VIP Database were searched from inception to December 2023, in Chinese and English. The search strategies were applied to each database using MeSH terms and natural language associated with the keywords “Patellar dislocation,” “MPFL,” “Single bundle,” and “Double bundle.” Table 1 shows the search strategy for the PubMed database.

**Table 1:** Search strategy for PubMed.

Query	Search term
#1	“Patellar Dislocation”[Mesh]
#2	“patellar dislocation”[Title/Abstract] OR “Dislocation, Patellar”[Title/Abstract] OR “Dislocations, Patellar”[Title/Abstract] OR “Patellar Dislocations”[Title/Abstract] OR “patellar instability”[Title/Abstract] OR “recurrent patellofemoral instability”[Title/Abstract] OR
#3 #1 OR #2	“Recurrent patellar dislocation”[Title/Abstract]
#4	“MPFL” [Title/Abstract] OR “MPFLR” [Title/Abstract] OR “Medial patellofemoral ligament”[Title/Abstract] OR “Medial patellofemoral ligament reconstruction”[Title/Abstract] OR “MPFL reconstruction”[Title/Abstract]
#5	“single bundle”[Title/Abstract] OR “double bundle[Title/Abstract]” OR “SB[Title/Abstract] OR “DB”[Title/Abstract] OR “double tunnel”[Title/Abstract] OR “single tunnel”[Title/Abstract] OR “ST”[Title/Abstract] OR “DT”[Title/Abstract] OR “single strand”[Title/Abstract] OR “double strand”[Title/Abstract] OR “SS”[Title/Abstract] OR “DS”[Title/Abstract] OR “single”[Title/Abstract] OR
#6	#3 AND #4 AND #5“double”[Title/Abstract]

### Inclusion and exclusion criteria

Inclusion criteria: (1) Population: skeletally mature, sex, and side‐unlimited, with a clear diagnosis of RPD and the need for MPFL reconstruction; (2) Study type: controlled trials (randomized controlled trials [RCTs], retrospective studies, prospective studies); (3) Interventions: a direct comparison of SB and DB reconstruction; (4) Outcome: knee function scores, such as those of Kujala, Lysholm, imaging indicators assessment, and postoperative complications.

Exclusion criteria: (1) Presence of severe injury to other structures (cruciate ligament, meniscus) other than MPFL injury; (2) case reports, cadaveric experiments, related reviews, conferences, dissertations; (3) MPFL reconstruction with a single or double bundle, concomitant tibial tubercle osteotomy, and/or trochleoplasty were reported; and (4) unavailable outcome data.

### Data extraction

The data were extracted independently by two researchers (Wu and Wang), and in the case of a disagreement, the third senior researcher (Zhao) was consulted for participation and resolution of the same disagreement. The extracted data were recorded and shown in Excel software in the form of three‐line tables. Extracted data: characteristics of studies included in the literature (first author, year of publication, type of study, outcome indicators), baseline patient information (sample size, gender, mean age, mean follow‐up time).

### Risk of bias assessment

The same two researchers did a quality assessment of the final included literature. The disagreements were referred to the third senior researcher for participation in the same disagreement. The Newcastle‐Ottawa Scale (NOS) [36] was chosen for the non‐RCTs, which evaluates three aspects: study population (SELECTION), between‐group comparability (COMPARABILITY), and outcome (OUTCOME). The higher the score, the higher the quality of the study. Less than 3 is classified as a low‐quality study, 4–6 as a moderate‐quality study, and 7–9 as a high‐quality study.

RCTs are evaluated using a quality risk bias evaluation tool recommended by the Cochrane Collaboration Network. The evaluation tool sheet includes seven aspects: (1) Random sequence generation; (2) Allocation concealment; (3) Performance bias; (4) Detection bias; (5) Completeness of outcome data; (6) Reporting bias; (7) Other bias.

### Statistical analysis

Extracted data were meta‐analyzed using the Review Manager 5.3 software provided by the Cochrane collaboration and Stata 16.0. Dichotomous variables were expressed as odds ratio (OR) and 95% confidence intervals (95% CI). Continuous variables were expressed as mean differences (MD) and 95% CI. *I* ^2^ was calculated to test the heterogeneity between different studies, when *I* ^2^ < 50%, it suggests that there is homogeneity between studies and fixed effect model was used; if *I* ^2^ > 50%, it suggests that there is heterogeneity between studies and random effect model was used. Subgroup analyses were conducted to explore sources of heterogeneity by study type, follow‐up time, and total knees. Sensitivity analysis is used to test the robustness of Meta‐analyses from heterogeneity studies. Publication bias was analyzed only for outcomes that included more than 10 studies. Egger's test and Begg's test were adopted to evaluate the publication bias. All tests were two‐sided and *p* < 0.05 was supposed to have a statistical significance.

## RESULT

### Research results

**Figure 1:**
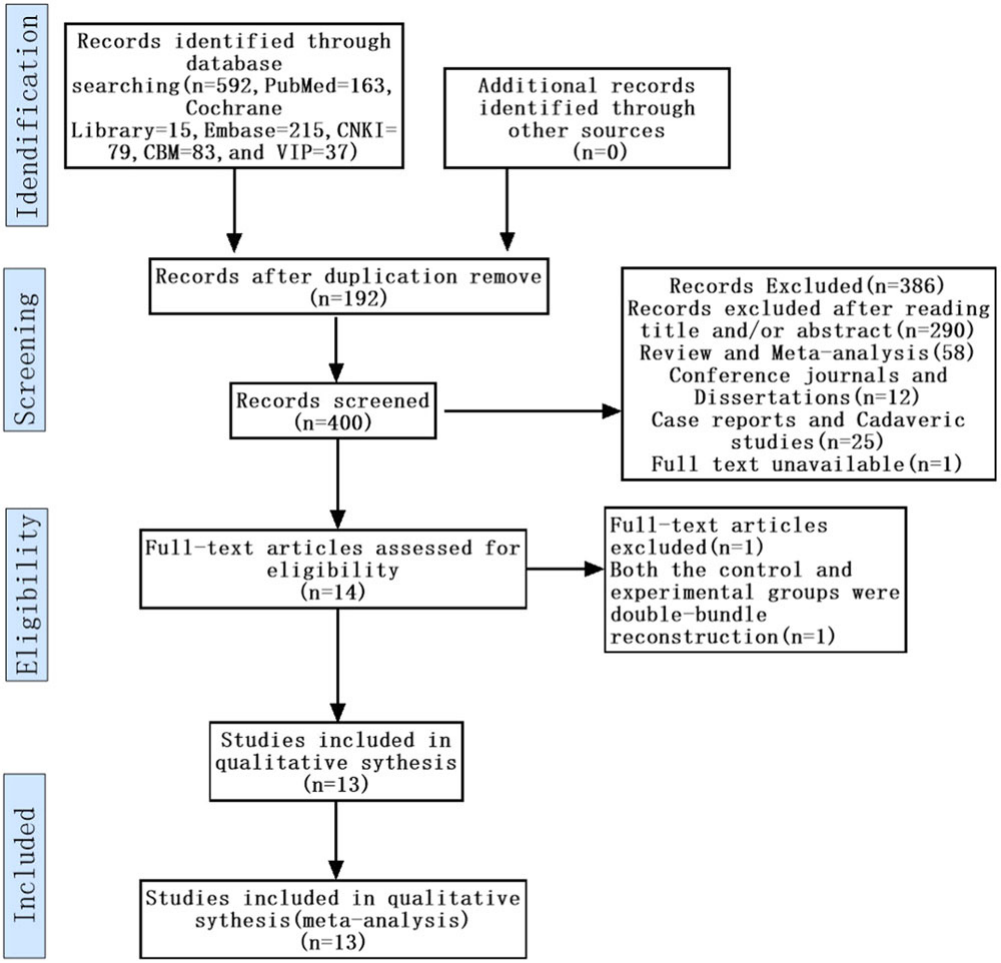
The flow chart of the study selection process.

A total of 592 papers were included in the screening, 192 duplicates were excluded, 95 reviews, conference journals, dissertations, case reports, cadaveric, and one full‐text unavailable study were excluded, 290 papers were excluded by reading the title abstracts, one paper failed to meet the criteria for inclusion or exclusion and 13 papers were finally included after reading the full text and according to the inclusion and exclusion criteria. Although Mohammed et al. [22] hadn^’^t described follow‐up time, some results were reported. Therefore, the article was included. The literature screening process is shown in Figure 1. The basic characteristics of the included studies and baseline patient information are shown in Table 2.

**Table 2:** Characteristics of the included studies and baseline patient information.

Author	Year	Patients (knees)		Sex (male/female)		Age (year)		Follow‐up time (months)		Research type	Level of evidence
		DB	SB	DB	SB	DB	SB	DB	SB		
Astur et al. [3]	2015	28 (28)	30 (30)	30/28		28.3 (18–45)	31.1 (18–45)	60		RCT	Ⅰ
Ercan et al. [7]	2021	40 (40)	40 (40)	18/22	20/20	19 (14–29)	15 (10–28)	40 (24–74)	46.5 (24–74)	RCT	Ⅰ
Gao et al. [9]	2015	17 (17)	12 (12)	11/18		24 (15–36)		14		CS	Ⅲ
Guo et al. [10]	2018	50 (50)	44 (44)	32/18	26/18	49.5 ± 9.3	49.6 ± 9.2	12		CS	Ⅲ
Li et al. [18]	2018	25 (25)	22 (22)	10/15	7/5	16–30	16–33	39		CS	Ⅲ
Li et al. [19]	2019	45 (45)	43 (43)	15/30	17/26	26.9 ± 5.4	27.4 ± 5.6	40.9 ± 7.2	41.3 ± 7.5	RCT	Ⅰ
Liu et al. [20]	2018	36 (36)	33 (33)	8/28	7/26	17.9 ± 4.3	18.5 ± 3.5	24 ± 2.1		CS	Ⅲ
Mohammed et al. [22]	2017	26 (29)	27 (29)	8/18	12/15	22.8 ± 8.5	20.9 ± 7.7	Undescribed		CS	Ⅲ
Qiao et al. [28]	2022	42 (42)	48 (48)	15/27	16/32	21.4 (14–41)	23.8 (15–39)	38.6 (25–53)	37.8 (27–50)	RCT	Ⅰ
Sun et al. [30]	2016	38 (38)	40 (40)	12/26	16/24	20.5 ± 3.3	20.2 ± 3.7	12 (10–14)		CS	Ⅲ
Wang et al. [32]	2013	37 (44)	21 (26)	16/21	7/14	26 ± 7	23 ± 10	12 and 48		CS	Ⅲ
Wang et al. [33]	2010	38 (45)	22 (27)	10/15	7/15	16–30	16–33	39		CS	Ⅲ
Wu et al. [35]	2017	26 (26)	32 (32)	7/19	10/22	21.8 (16–28)	22.5 (16–28)	23.9 (18–30)		CS	Ⅲ

Abbreviations: DB, double‐bundle; RCS, retrospective cohort study; RCT, randomized controlled trials; SB, single‐bundle.

### Results of the quality assessment

**Figure 2:**
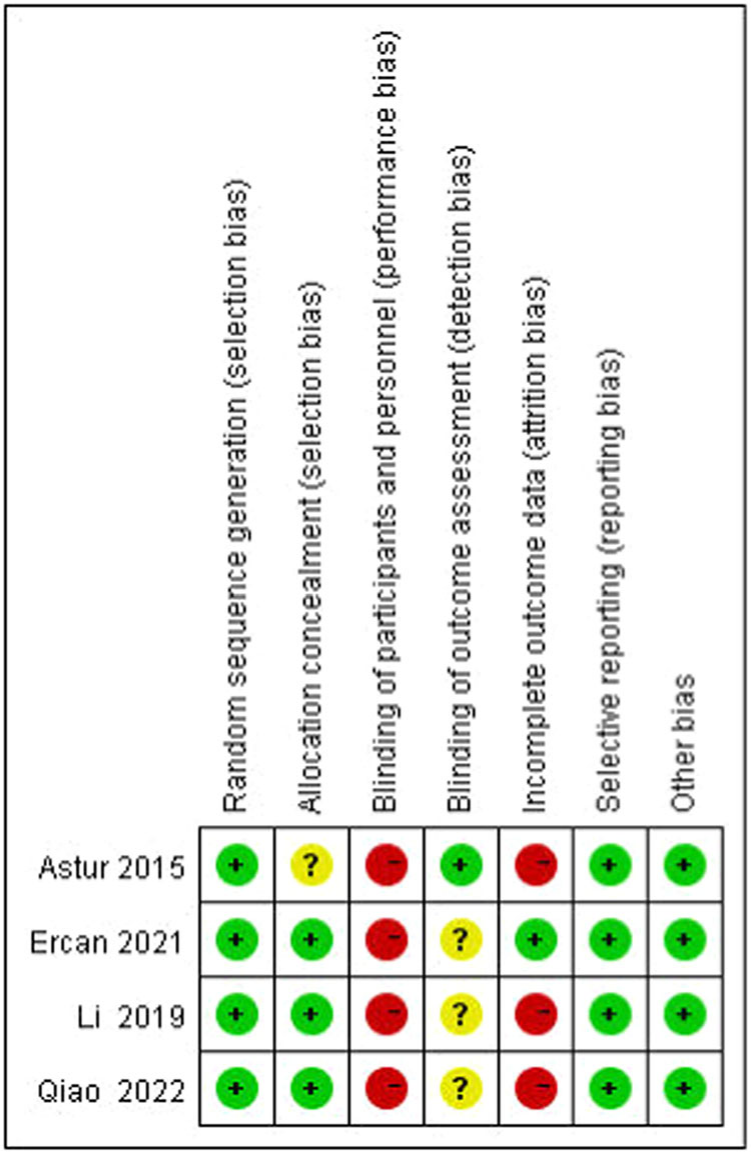
Risk of bias summary.

**Figure 3:**
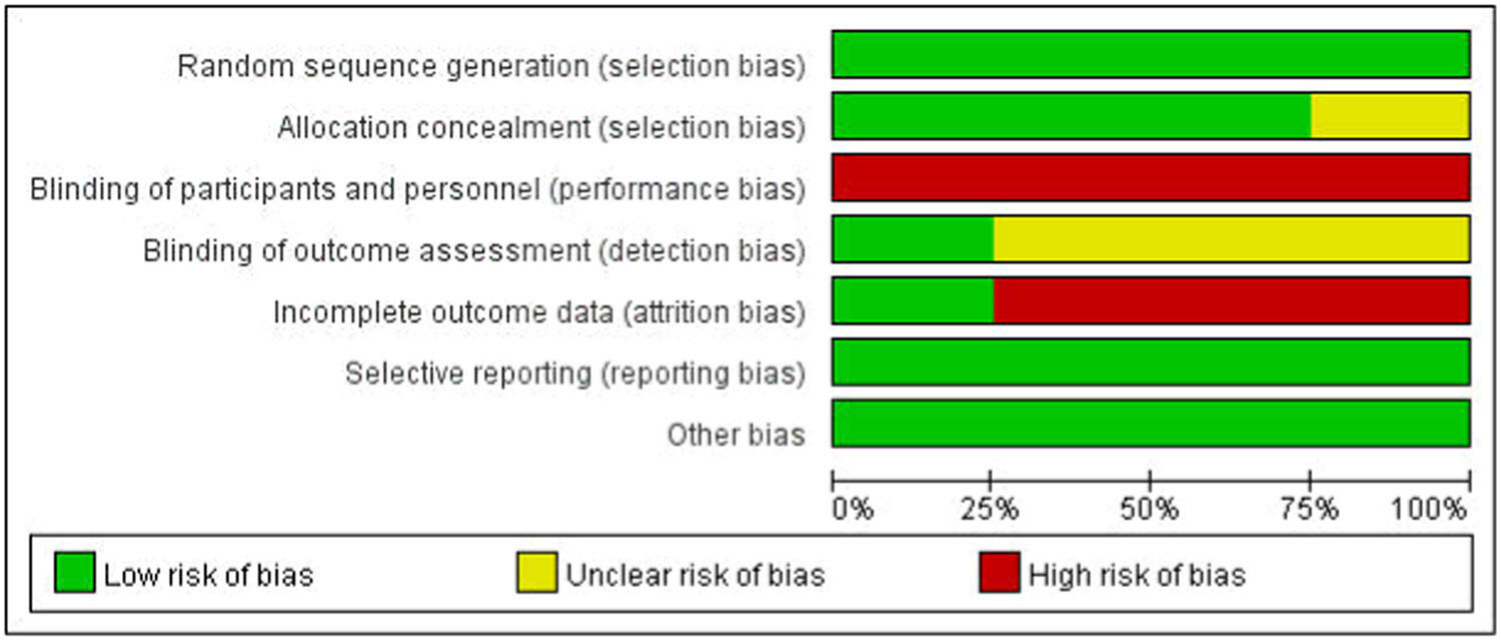
Risk of bias graph. Red for high risk, green for low risk, and yellow for unclear risk of bias.

Of the 13 papers included, four [3, 7, 19, 28] were RCTs and nine [9, 10, 18, 20, 22, 23, 30, 33, 35] were Cohort studies (CSs). A total of 862 (891 knees) patients were included, including 448 (465 knees) with DB reconstruction and 414 (426 knees) with SB reconstruction. Of the cohort studies, one scored 5, one scored 6, and seven scored 7 (Table 3). Four RCTs were studied for their risk of bias as shown in Figures 2 and 3.

**Table 3:** The Newcastle‐Ottawa Scale (NOS).

Study	Selection	Comparability	Outcome	Total stars	Quality
Gao et al. [9]	✩✩✩	✩✩	✩✩	7	High
Guo et al. [10]	✩✩	✩✩	✩✩	6	Moderate
Li et al. [18]	✩✩✩	✩✩	✩✩	7	High
Liu et al. [20]	✩✩✩	✩✩	✩✩	7	High
Mohammed et al. [22]	✩✩✩	✩✩	—	5	Low
Sun et al. [30]	✩✩✩	✩✩	✩✩	7	High
Wang et al. [32]	✩✩✩	✩✩	✩✩	7	High
Wang et al. [33]	✩✩✩	✩✩	✩✩	7	High
Wu et al. [35]	✩✩✩	✩✩	✩✩	7	High

### Meta‐analysis results

#### Kujala Score

**Figure 4:**
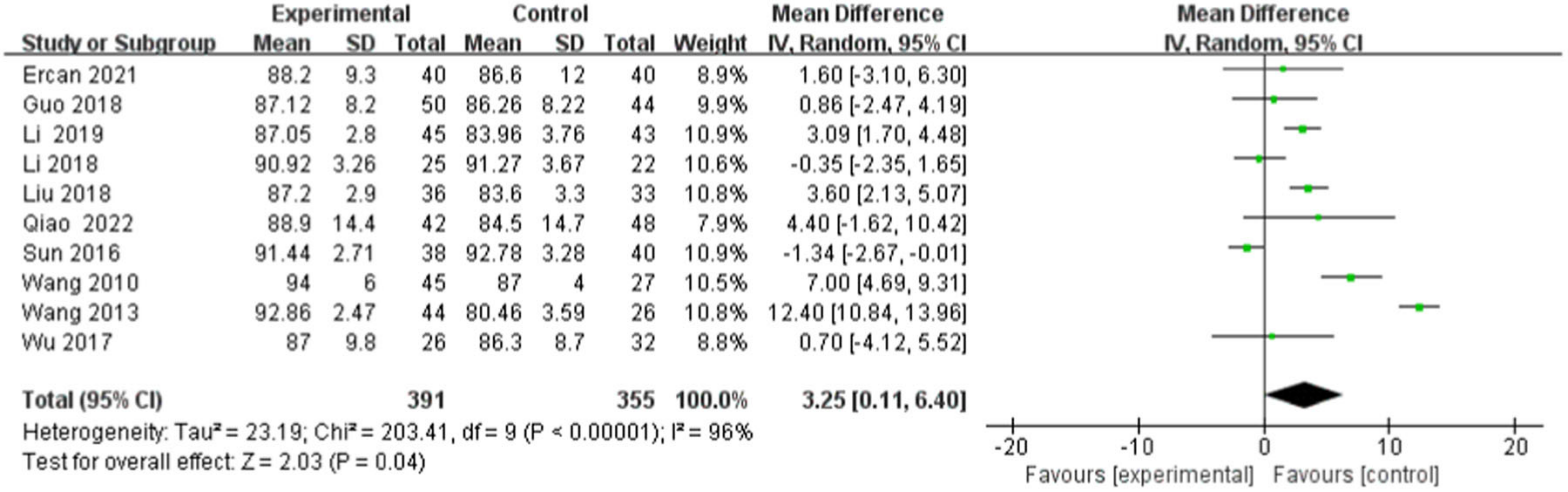
Forest plot of the comparison of the Kujala score between the double‐bundle (DB) and single‐bundle (SB).

Ten studies [7, 10, 18, 19, 20, 28, 30, 32, 33, 35] compared postoperative Kujala scores, 377 (391 knees) patients in the DB reconstruction group and 345 (355 knees) patients in the SB reconstruction group. The heterogeneity test suggested heterogeneity among studies (*I* ^2^ = 84%, *p* < 0.01), so a random effects model was used. Results showed that the Kujala score in the DB reconstruction group was better than in the SB reconstruction group, and the difference was statistically significant (MD = 2.06, 95% CI [0.11, 4.01], *p* < 0.05) (Figure 4).

#### Lysholm Score

**Figure 5:**
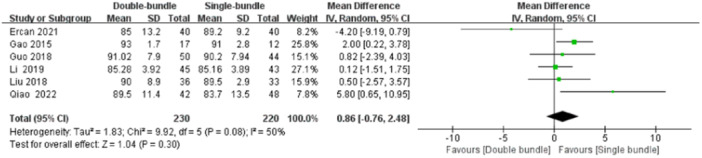
Forest plot of the comparison of the Lysholm score between the double‐bundle (DB) and single‐bundle (SB).

Six studies [7, 9, 10, 19, 20, 28] compared postoperative Lysholm scores, 230 (230 knees) patients in the DB group and 220 (220 knees) patients in the SB group. The heterogeneity test suggested heterogeneity between studies (*I* ^2^ = 50%, *p* = 0.08), so a random effects model was used. The results showed no statistically significant difference between the two groups (MD = 0.86, 95% CI [−0.76, 2.48], *p* = n.s], Figure 5.

#### Tegner Score

**Figure 6:**
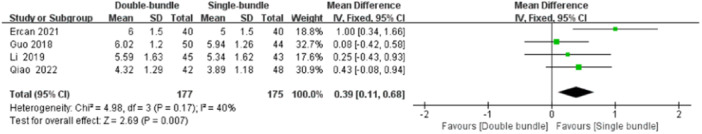
Forest plot of the comparison of the Tegner score between the double‐bundle (DB) and single‐bundle (SB).

Four studies [7, 9, 19, 20] compared postoperative Tegner scores in 177 (177 knees) patients in the DB group and 175 (175 knees) patients in the SB group. The heterogeneity test suggested homogeneity among the studies (*I* ^2^ = 40%, *p* = 0.17), so a fixed‐effects model was used. The results showed that the DB reconstruction Tegner score was improved to the SB reconstruction Tegner score, and the difference was statistically significant (MD = 0.39, 95% CI [0.11, 0.68], *p* < 0.05], Figure 6.

### International Knee Documentation Committee (IKDC) Score

**Figure 7:**
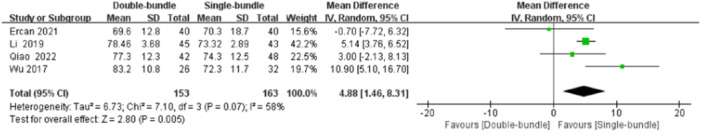
Forest plot of the comparison of the International Knee Documentation Committee (IKDC) score between the double‐bundle (DB) and single‐bundle (SB).

Four studies [7, 19, 28, 35] compared postoperative IKDC scores, 153 (153 knees) patients in the DB group and 163 (163 knees) patients in the SB group. The heterogeneity test suggested heterogeneity among studies (*I* ^2^ = 58%, *p* = 0.07), so a random‐effects model was used. The results indicated that the DB reconstruction IKDC score was superior to the SB reconstruction IKDC score, and the difference was statistically significant (MD = 4.88, 95% CI [1.46, 8.31], *p* < 0.05), Figure 7.

#### Patellar tilt angle (PTA)

**Figure 8:**
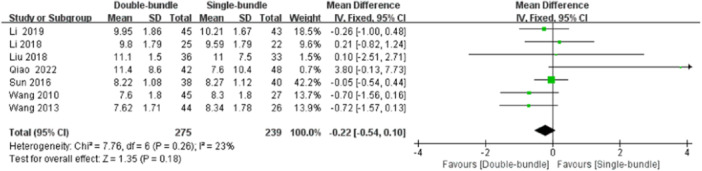
Forest plot of the comparison of the Patellar Tilt Angle (PTA) between the double‐bundle (DB) and single‐bundle (SB).

The patellar tilt angle was measured using CT in all the studies. The heterogeneity test suggested heterogeneity between studies (*I* ^2^ = 23%, *p* = 0.26), so a fixed‐effects model was used. The results showed no statistically significant difference between the two groups (MD = −0.22, 95% CI [−0.54, 0.10], *p* = n.s), Figure 8.

#### Congruence angle (CA)

**Figure 9:**
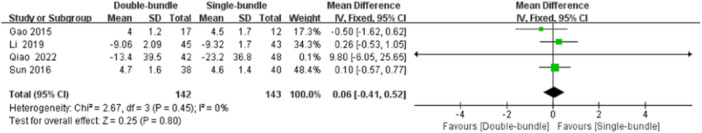
Forest plot of the comparison of the congruence angle (CA) between the double‐bundle (DB) and single‐bundle (SB).

Four studies [9, 19, 28, 30] compared postoperative patellofemoral congruence angle, 142 (142 knees) patients in the DB group and 143 (143 knees) patients in the SB group. The CA was measured using CT in all the studies. The heterogeneity test suggested homogeneity among the studies (*I* ^2^ = 0%, *p* = 0.45), so a fixed‐effects model was used. The results showed no statistically significant difference in the patellofemoral fit angle between the two groups (MD = 0.06, 95% CI [−0.41, 0.52], *p* = n.s), Figure 9.

#### Patellar lateral shift rate (PLSR)

**Figure 10:**
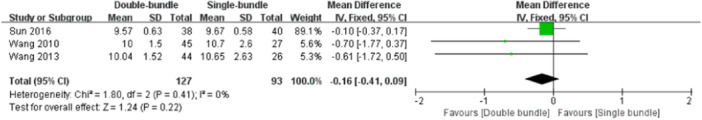
Forest plot of the comparison of the patellar lateral shift rate (PLSR) between the double‐bundle (DB) and single‐bundle (SB).

Three studies [30, 32, 33] compared the postoperative patellar lateral shift rate in 113 (127 knees) patients in the DB group and 83 (93 knees) patients in the SB group. The PLSR was measured using CT in all the studies. The heterogeneity test suggested heterogeneity between studies (*I* ^2^ = 0%, *p* = 0.41), so a fixed effects model was used. The results showed no statistically significant difference when comparing the patellar lateral shift rate between the two groups (MD = −0.16, 95% CI [−0.41, 0.09], *p* = n.s), Figure 10.

#### Postoperative Instability

**Figure 11:**
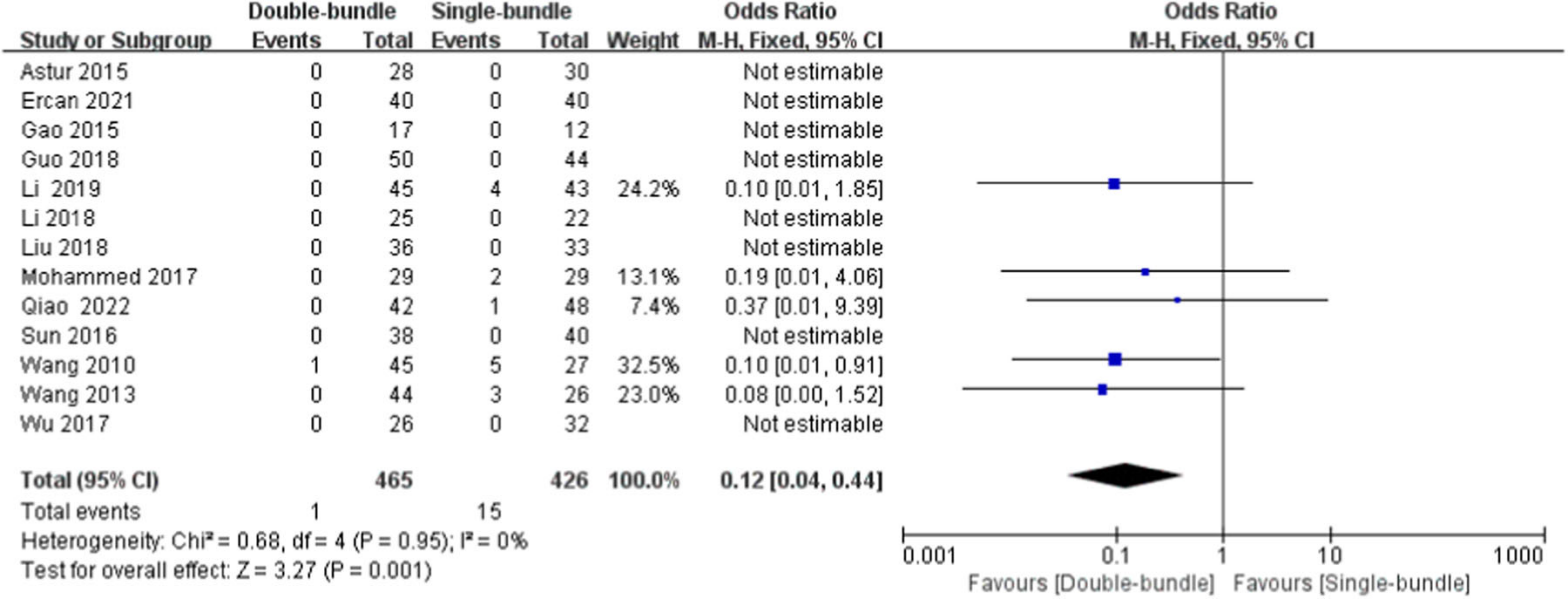
Forest plot of the comparison of the postoperative instability between the double‐bundle (DB) and single‐bundle (SB).

Postoperative recurrent instability was described in 13 studies [3, 7, 9, 10, 18, 19, 20, 22, 28, 30, 32, 33, 35], with 448 (465 knees) patients in the DB group and 414 (426 knees) patients in the SB group. Postoperative recurrent patellar instability can be described on the clinician's physical examination and the appearance of the dislocation. In 16 patients with postoperative recurrent instability, the heterogeneity test demonstrated homogeneity in the study (*I* ^2^ = 0%, *p* = 0.95), and therefore a fixed‐effect model was used. The results showed that the incidence of recurrent instability after DB reconstruction was lower than that in the SB reconstruction group, and the difference was statistically significant (OR = 0.12, 95% CI [0.04, 0.44], *p* < 0.05) (Figure 11).

#### Postoperative knee pain

**Figure 12:**
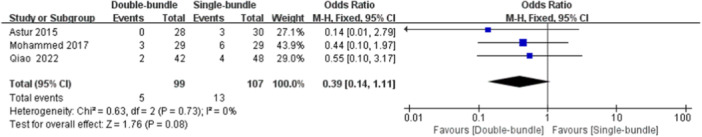
Forest plot of the comparison of the postoperative pain in one knee between the double‐bundle (DB) and single‐bundle (SB).

Three studies [3, 22, 28] compared postoperative knee pain, 96 (99 knees) patients in the DB group, and 105 (107 knees) patients in the SB group. The heterogeneity test suggested the homogeneity among the studies (*I* ^2^ = 0%, *p* = 0.73), so a fixed‐effects model was used. Results showed no statistically significant difference in the incidence of postoperative pain in one knee between the two groups (OR = 0.39, 95% CI [0.14, 1.11], *p* = n.s), Figure 12.

#### Additional postoperative surgical treatment

**Figure 13:**
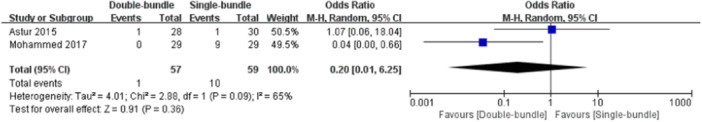
Forest plot of the comparison of the additional postoperative surgical treatment between the double‐bundle (DB) and single‐bundle (SB).

Two studies [3, 22] compared additional postoperative surgical treatment, 54 (57 knees) patients in the DB group and 57 (59 knees) patients in the SB group. When postoperative complications occur (patellar fracture, postoperative arthrofibrosis, etc.) additional postoperative surgical treatment is required. The heterogeneity test suggested heterogeneity between studies (*I* ^2^ = 65%, *p* = 0.09), so a random‐effects model was used. The results showed no statistically significant difference in the need for additional postoperative surgical treatment between the two groups (OR = 0.20, 95% CI [0.01, 6.25], *p* = n.s) (Figure 13).

### Subgroup analysis and sensitivity analysis

**Figure 14:**
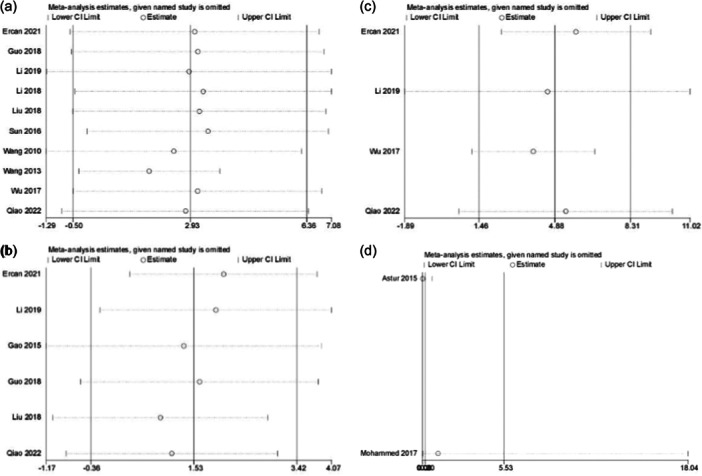
Sensitivity analysis results. (a) Kujala score; (b) Lysholm score; (c) International Knee Documentation Committee (IKDC) score; (d) additional postoperative surgical interventions.

The Kujala, Lysholm, and IKDC score heterogeneity tests indicated the presence of heterogeneity. Subgroup analyses were performed to explore the source of heterogeneity based on study type (RCT, CS), follow‐up time (greater than 40 months, less than 40 months), knees, and the results of the analyses suggested that study type, follow‐up time, and knees were not sources of heterogeneity (Table 4). Sensitivity analyses of the Kujala score, Lysholm score, IKDC score, and additional postoperative surgical treatment were performed to test the robustness of the results (Figure 14). The sensitivity analyses of the Kujala score, Lysholm score, and IKDC score did not significantly affect the results whenever a study was excluded, so the results of the Meta‐analysis were stable. In the sensitivity analysis of additional postoperative surgical treatment, the exclusion of Astur's [3] study had a significant effect on the results, and thus the meta‐analysis was not stable.

**Table 4:** Results of the subgroup analysis.

Outcome	Factors	Subgroup	MD (95% CI)	*I* ^2^ (%)	*p* (Heterogeneity)	*p*‐value
Kujala score	Follow‐up time	>40 months	3.04 (1.74, 4.34)	0	=0.76	< 0.01
		<40 months	1.74 (−0.85, 4.34)	88	<0.01	=0.19
	Research type	RCT	3.04 (1.74, 4.34)	0	=0.76	<0.01
		CS	1.74 (−0.85, 4.34)	88	<0.01	=0.19
	Knees	≥80	2.75 (1.54, 3.96)	0	=0.57	<0.01
		<80	1.88 (−1.05, 4.81)	90	<0.01	=0.21
Lysholm score	Follow‐up time	>40 months	−1.37 (−5.40, 2.65)	62	=0.11	=0.50
		<40 months	2.15 (0.23, 4.06)	23	=0.27	=0.03
	Research type	RCT	0.47 (−3.87, 4.81)	74	=0.02	=0.83
		CS	2.53 (1.05, 4.01)	40	=0.19	<0.01
IKDC score	Knees	≤80	5.28 (−6.09, 16.64)	84	=0.01	=0.36
		>80	5.00 (3.66, 6.33)	0	=0.43	<0.01

Abbreviations: CI, confidence intervals; CS, cohort studies; IKDC, International Knee Documentation Committee; MD, mean differences; RCT, randomized controlled trials.

### Publication bias

**Figure 15:**
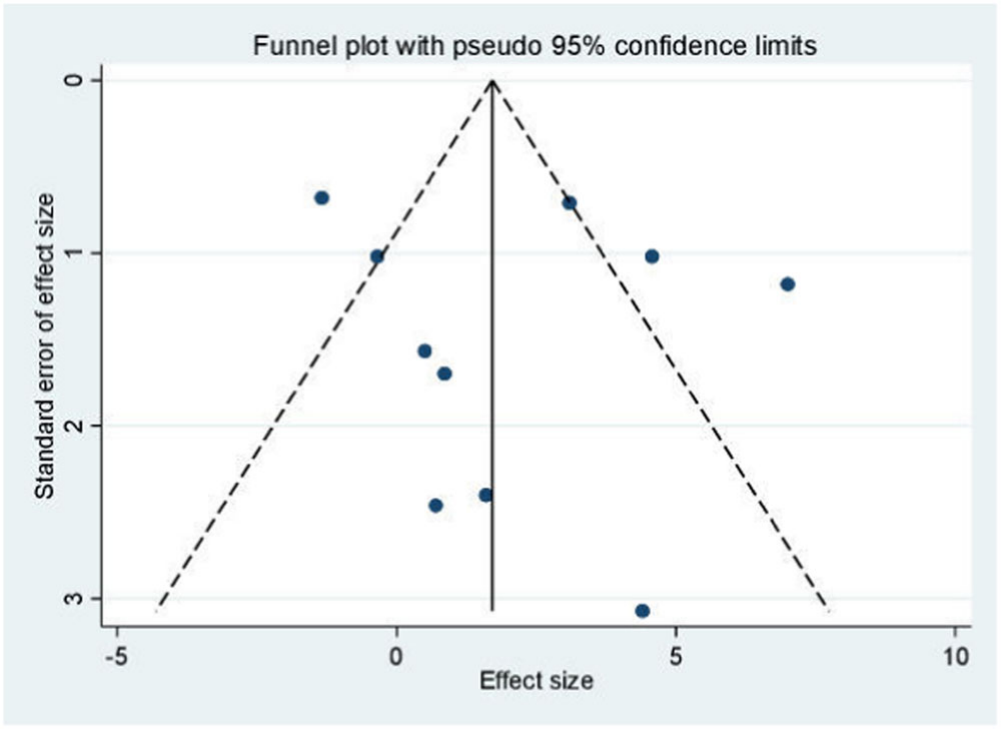
Funnel plots of the Kujala score.

**Figure 16:**
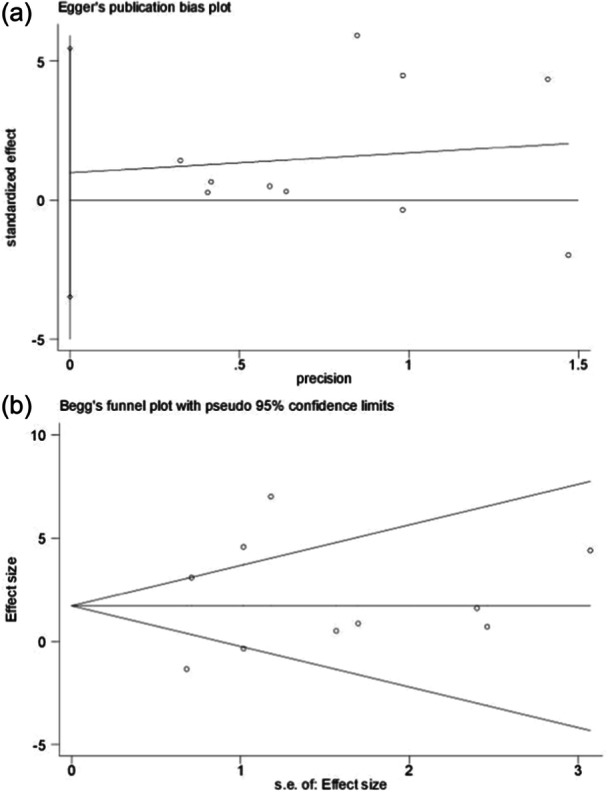
The results of publication bias. (a) Egger's test; (b) Begg's test.

The principle of publication bias analysis is to include at least 10 studies. Only the Kujala score was analyzed for publication bias. Funnel plots of studies included in the Kujala score are shown in Figure 15. Egger's test (*p* = 0.623) and Begg's (*p* = 0.474) in Figure 16a,b came up with nonsignificant results, implying no publication bias.

## DISCUSSION

The SB reconstruction was inferior to DB reconstruction in the incidence of postoperative recurrent instability and the postoperative Kujala, Tegner, and IKDC scores. Concerning the Lysholm score, patellar tilt angle, congruence angle, patellar lateral shift rate, postoperative knee pain, and additional postoperative surgical treatment, there was no statistically significant distinction between the two groups.

The DB reconstruction showed superior Kujala, Tegner, and IKDC scores compared to the SB reconstruction, which is consistent with the conclusions of the Pang et al. [24] meta‐analysis. The knee function scores of DB reconstruction are greater than those of SB reconstruction, according to the findings from many research articles [21, 32, 33]. However, the study by Heo et al. [11] found that patients who underwent SB reconstruction had higher Kujala scores. During the three processes of necrosis, neovascularization, and remodeling that reconstructed tendon experience, ligament strength diminishes [32]. The DB isometric reconstruction of the MPFL may not offer adequate traction during the early rehabilitation stage, yet a DB anatomic reconstruction can give twice as much traction [32]. Consequently, the ligament is less prone to stretching during the initial phase of rehabilitation, leading to improved knee functionality [32].

Imaging assessment fails to show any distinction between SB and DB reconstruction procedures. Accordingly, all techniques are equally effective in restructuring the patellofemoral joint. The measurement of the patellar tilt angle was conducted by matching two planes that overlapped the longest axis of the patella and the “Roman arch” of the inter‐condylar fossa [1, 5, 37]. Furthermore, several researchers have measured the angle of patellar tilt using only one plane and using the longest axis of the patella [2, 31]. The selection of the level of the femoral condyles in the shape of a “Roman arch” is subjective, and differences in measurement methods can cause bias in the results. The patellar tilt angle can be measured in the axial position using CT or MRI. The patellar tilt angle of this meta‐analysis was all measured at CT. It has been suggested that the axial position is not suitable for measuring the congruence angle due to changes in knee position during the examination or difficulty in recognizing the lowest point of the patellar articular surface [38]. Since there are no differences in the patellar tilt angle, congruence angle, or patellar lateral shift rate between the two groups, it has been proposed that DB isometric reconstruction is unable to fully restore the normal patellar track at any knee flexion angle. On the other hand, DB anatomical reconstruction is unable to completely restore the normal patellar track, but it can maintain the normal patellar track in the center of the trochlear groove [25]. Abnormal patellar trajectory can result in abnormal stress distribution, which leads to articular cartilage injury and patellofemoral pain. Thus, a longer follow‐up is needed to compare whether there is a difference between the two.

The incidence of postoperative recurrent instability was significantly lower in the DB reconstruction than in the SB reconstruction group. In contrast, there was no difference in postoperative peripatellar pain or the need for additional postoperative surgical intervention. This result is the same as the conclusion of Pang et al. [24] the incidence of postoperative recurrent patellar instability was lower in the DB group than in the SB reconstruction group. Lee et al. [16] performed a meta‐analysis of surgical techniques for patellofemoral instability. They reviewed two clinical trials comparing DB versus SB and found reduced instability, revisions, and improved clinical score outcomes in the DB group. However, a systematic review by Kang et al. [13] reported similar results between the two reconstruction groups in terms of postoperative recurrent dislocation and complication rates. Three patients in the DB group experienced patellar re‐dislocation, no re‐dislocations occurred in the DB group in the study by Wang et al. [32]. The DB reconstruction creates an angle of 12°–15° between the two bundles, which has a synergistic effect. Additionally, the DB reconstruction mimics the anatomical structure of the MPFL, requiring greater resistance against patellar translation before it enters the femoral trochlea. Hence, in the case of a 15° flexed knee, a DB MPFL reconstruction requires more force compared to an SB MPFL reconstruction to induce a 10 mm lateral displacement [34]. Moreover, the DB reconstruction not only reduced patellar rotation during flexion and extension movements, which can happen with SB reconstruction, but it also demonstrated superior load‐bearing capacity (with a limiting load of 213 ± 90 N compared to 171 ± 51 N for the DB group) and more balanced distribution of physiological stress [26, 29]. From a biomechanical perspective, DB reconstruction restores the biomechanical function of the MPFL to the greatest degree possible, so there is a low incidence of recurrent instability in the postoperative period.

## STUDY LIMITATIONS

(1) The quantity of literature included was moderate and of average quality. None of the four randomized controlled studies included were double‐blind, and three had patients lost to follow‐up, but the rate of loss to follow‐up was very low. One cohort study was of low quality and another cohort study was of moderate quality. (2) The indicators of postoperative pain in one knee, additional postoperative surgical treatment, and patellar lateral shift rate were included in a smaller number of studies, which affects the accuracy of the test results. (3) Heterogeneity of the Kujala score scores, Lysholm scores, and IKDC score studies has not been identified. Nine of the studies involved patients in China, which may affect the reliability and accuracy of results based on race.

## CONCLUSIONS

DB MPFL reconstruction for RPD was superior to SB reconstruction in both knee function scores and postoperative recurrent patellar instability, and MPFL reconstruction with DB for RPD had better clinical outcomes. However, due to the inherent limitations of the included RCTs and CSs, high‐quality clinical trials are still needed to further assess the effectiveness of SB versus DB MPFL reconstruction for the treatment of RPD.

## AUTHOR CONTRIBUTIONS

**Yiheng Wu**: Conceptualization; data curation; formal analysis; investigation; methodology; writing—original draft. **Junran Li**: Methodology; conceptualization; supervision; writing review and editing. **Hongbo Zhao**: Methodology; conceptualization; project administration; writing review and editing. **Hongyan Zhou**: Methodology; conceptualization; writing review and editing. **Bokai Wang**: Data curation. **Jinlong Zhang**: Data curation. **Shengkun Zhao**: Data curation.

## CONFLICT OF INTEREST STATEMENT

The authors declare no conflicts of interest.

## ETHICS STATEMENT

There was no ethical approval because this study was a meta‐analysis based on the data of previously published studies.

## Data Availability

The databases analyzed during the current study are available.
